# Incidence and Risk of Secondary Malignancy in Patients with Waldenström Macroglobulinemia: A Population-Based Analysis

**DOI:** 10.46989/001c.90436

**Published:** 2024-01-05

**Authors:** Mohammad Ebad Ur Rehman, Maha Hameed, Zunairah Shah, Omer S Ashruf, Rabia Ali, Fatima Faraz, Jawad Basit, Israr Khan, Faizan Fazal, Ahmad Iftikhar, Abdulqadir J Nashwan, Muhammad Salman Faisal, Faiz Anwer

**Affiliations:** 1 Rawalpindi Medical University; 2 Department of Internal Medicine Sarasota Memorial Hospital; 3 Department of Internal Medicine Weiss Memorial Hospital; 4 College of Medicine Northeast Ohio Medical University; 5 Department of Internal Medicine University of Arkansas for Medical Sciences; 6 Department of Internal Medicine Hackensack Meridian Health; 7 Department of Medicine University of Arizona; 8 Hamad Medical Corporation; 9 Division of Hematology Roswell Park Cancer Institute; 10 Department of Hematology and Medical Oncology Cleveland Clinic

**Keywords:** Waldenström Macroglobulinemia, Cancer Recurrence, Hematological Malignancy, Plasma Cell Dyscrasia, Risk Stratification

## Abstract

Waldenström macroglobulinemia (WM) is a rare lymphoplasmacytic lymphoma which may predispose individuals to development of secondary malignancies (SMs). The Surveillance, Epidemiology, and End Results (SEER) database is a comprehensive registry of cancer patients in the United States reporting on a wide set of demographic variables. Using the SEER-18 dataset, analyzing patients from 2000 to 2018, we aimed to assess the incidence of SMs in WM patients. Patient characteristics such as gender, age, race, and latency were identified, and respective standardized incidence ratios (SIRs) and absolute excess risks (AERs) were calculated to compare to the general population. Of the 4,112 eligible WM patients identified, SMs were reported in 699 (17%) patients. The overall risk of developing SM, second primary malignancy, and secondary hematological malignancy was significantly higher in WM patients compared to the general population. Our findings show that WM patients had a 53% higher risk of SMs relative to the general population, and an AER of 102.69 per 10,000. Although the exact mechanism is unclear, the risk of SM development may be due to genetic predisposition, immune dysregulation, or treatment-induced immune suppression.

## Introduction

Waldenström macroglobulinemia (WM) is an indolent B-cell neoplasm manifesting as monoclonal gammopathy and bone marrow infiltration of plasma cells.^1^ It remains an incurable disease with disease-specific mortality of nearly 25%. However, most patients have prolonged survival.^2^

Small studies have shown an increased risk of secondary malignancies (SMs) in patients with WM, given its prolonged survival and immune dysregulation.^3–5^ However, few studies compare the incidence of SMs in WM with that of the general population. An analysis of the Surveillance, Epidemiology, and End Results (SEER) database in 2015 demonstrated that patients with WM have a 49% increased risk of SMs compared to the general population.^6^ A literature review has shown an increased risk of hematological malignancies such as acute myeloid leukemia, aggressive diffuse large B-cell lymphoma, and Burkitt lymphoma.^5,7–9^ The exact mechanism of the development of SMs in WM is unclear; various hypotheses have been proposed, including genetic predisposition, immune dysregulation, or treatment targeting related SMs.

We aimed to analyze the incidence of SM in patients with WM, focusing on specific patient characteristics and comparing them with the general population.

## Methods

This retrospective study used patient data from the Surveillance, Epidemiology, and End Results (SEER) database. The SEER-18 dataset is a repository of records covering approximately 27.8% of the U.S. population, reporting an aggregate of cases from 2000 to 2018. The SEER Program provides data on cancer diagnosis, mortality, incidence, treatment, prevention, and respective demographic variables.

We identified all patients diagnosed with WM, per the International Classification of Diseases in Oncology, Third Edition [ICD-O-3] code 9761/3, from 2000 to 2018. We further refined the inclusion criteria by incorporating SM diagnoses, defined as a separate malignancy appearing at least a year after the diagnosis of WM. Patients diagnosed with WM via autopsy, death certificate, or with incomplete follow-up data were excluded from the analysis. Latency was also assessed as the interval between WM and SM diagnoses. Using the SEER*Stat software (version 8.3.9), standardized incidence ratios (SIRs) and absolute excess risks (AERs) were calculated.

### Statistical Analysis

Gender, age, race, and latency were factors used to stratify the relationship and risk of SM in WM patients. SIRs were calculated to quantify SM risk and compare the incidence of SM in patients diagnosed with WM to that in the general population. AERs were also calculated to determine excess cancer rates in different cohorts, indicative of the clinical burden of additional cancer. P-values and 95% confidence intervals (CI) were generated assuming the Poisson distribution of the observed incidence of SMs.

## Results

We identified 4,112 eligible WM patients reported by the SEER-18 registries between 2000 and 2018. The average age for occurrence of SM was 74 years. Selected patient characteristics are shown in Table 1. SM was reported in 699 WM patients (17.0%). The overall risk of developing SM was significantly higher in WM patients compared to the general population (SIR= 1.53, 95% Cl=1.42-1.65, p <0.05) with an AER of 102.69 per 10,000 population.

**Table 1.** Demographic information of patients with secondary malignancy in Waldenström macroglobulinemia

***Demographic characteristics***		***N***
*Total number of patients with SM*		*4,112*
*Gender*		
	*Male*	*2,505*
	*Female*	*1,607*
*Race*		
	*Caucasian*	*3,746*
	*African American*	*178*
	***Other (American Indian/Alaska Native, Asian/Pacific Islander)***	*188*
*Person Years at Risk*		*23,625.72*
*Mean Person Years at Risk*		*5.75*
*Mean Age at Exposure*		*69.13*
*Mean Date of Exposure*		*2009.35*
*Mean Age at Event (for All Sites)*		*74*

SM: secondary malignancy

The risk of developing second primary solid tumors was higher than the general population (SIR= 1.12, 95% Cl= 1.02- 1.23, p <0.05) with an AER of 19.79. The overall risk of developing secondary hematological malignancies was also significantly increased (SIR= 4.95, 95% Cl 4.31-5.66, p <0.05) with an AER of 71.95. Table 2 illustrates the incidence of SM in WM patients.

**Table 2.** Locations of secondary malignancies in Waldenström macroglobulinemia patients

*Location*	*Observed*	*O/E*	*95% CI*	*Excess Risk*
*All sites*	*699*	1.53*	*1.42-1.65*	*102.69*
*All sites excluding Non-Melanoma Skin*	*688*	1.52*	*1.41-1.63*	*99.18*
*All Solid Tumors*	*436*	1.12*	*1.02-1.23*	*19.79*
*Floor of Mouth, Gum and Other Mouth*	*7*	3.28*	*1.32-6.76*	*2.06*
*Digestive System*	*64*	0.73*	*0.56-0.93*	*-10.11*
*Colon, Rectum and Anus*	*29*	0.65*	*0.44-0.94*	*-6.48*
*Colon and Rectum*	*28*	0.65*	*0.43-0.94*	*-6.31*
*Rectum and Rectosigmoid Junction*	*4*	0.37*	*0.1-0.94*	*-2.9*
*Rectum, Rectosigmoidal Junction, Anus, Anal Canal and Anorectum*	*5*	0.41*	*0.13-0.95*	*-3.07*
*Respiratory System*	*106*	1.44*	*1.18-1.74*	*13.68*
*Lung, Bronchus, Trachea, Mediastinum and Other Resp Org*	*100*	1.44*	*1.17-1.75*	*12.94*
*Lung and Bronchus*	*100*	1.44*	*1.17-1.75*	*12.98*
*Skin excluding Basal and Squamous*	*55*	2.10*	*1.59-2.74*	*12.22*
*Melanoma of the Skin*	*44*	1.88*	*1.36-2.52*	*8.7*
*Other Non-Epithelial Skin*	*11*	4.09*	*2.04-7.31*	*3.52*
*Breast*	*29*	*0.75*	*0.5-1.08*	*-4.08*
*Female Genital System*	*15*	*1.03*	*0.57-1.69*	*0.17*
*Male Genital System*	*84*	*1.09*	*0.87-1.35*	*3.01*
*Urinary System*	*47*	*0.98*	*0.72-1.3*	*-0.41*
*Eye and Orbit*	*0*	*0*	*0-5.01*	*-0.31*
*Brain and Other Nervous System*	*12*	2.63*	*1.36-4.6*	*3.15*
*Brain*	*12*	2.73*	*1.41-4.78*	*3.22*
*Endocrine System*	*7*	*1.38*	*0.56-2.85*	*0.82*
*All Lymphatic and Hematopoietic Diseases*	*213*	4.95*	*4.31-5.66*	*71.95*
*Lymphoma*	*170*	7.98*	*6.82-9.27*	*62.93*
*Non-Hodgkin Lymphoma (NHL)*	*167*	8.19*	*6.99-9.53*	*62.05*
*NHL - Nodal*	*90*	6.62*	*5.33-8.14*	*32.34*
*NHL - Extranodal*	*77*	11.30*	*8.92-14.12*	*29.71*
*Myeloma*	*14*	1.93*	*1.05-3.24*	*2.85*
*Leukemia*	*29*	2.01*	*1.35-2.89*	*6.17*
*Acute Lymphocytic Leukemia*	*3*	8.04*	*1.66-23.5*	*1.11*
*Non-Lymphocytic Leukemia*	*22*	3.02*	*1.89-4.57*	*6.23*
*Acute Non-Lymphocytic Leukemia (ANLL)*	*20*	4.20*	*2.56-6.48*	*6.45*
*Myeloid and Monocytic Leukemia*	*21*	3.28*	*2.03-5.01*	*6.18*
*Acute Myeloid Leukemia*	*18*	4.22*	*2.5-6.67*	*5.81*
*Mesothelioma*	*4*	*2.54*	*0.69-6.51*	*1.03*
*Kaposi Sarcoma*	*1*	*4.13*	*0.1-23.03*	*0.32*
*Miscellaneous*	*39*	1.82*	*1.29-2.49*	*7.43*

Secondary Malignancy Total, Persons at risk = 4112, Person years at risk = 23,625.72

Cl: confidence interval; O/E: observed/expected

* represents statistical significance

### Risk of SM at different sites

Among solid tumors, the risk was significantly increased for non-melanotic skin tumors (SIR 4.09) followed by mouth (SIR 3.28), brain (SIR 2.73), skin excluding basal and squamous carcinomas (SIR 2.10), skin melanoma (SIR 1.88), respiratory (SIR 1.44) and gastrointestinal cancer (SIR 0.73). The risk for hematological malignancies was also increased (SIR 4.95): extra-nodal non-Hodgkin lymphoma (NHL) (SIR 11.3), NHL (SIR 8.29), acute lymphocytic leukemia (ALL) (SIR 8.04), lymphoma (SIR 7.98), nodal NHL (SIR 6.62), acute myeloid leukemia (AML) (SIR 4.22), acute non-lymphocytic leukemia (ANLL) (SIR 4.2), myeloid and monocytic leukemia (SIR 3.28), leukemia (SIR 2.01), and myeloma (SIR 1.93) as reported fully in Table 2.

### Risk of SM stratified by latency

Of patients who developed SM 12-59 months after WM diagnosis, the greatest risks were seen for nonepithelial skin cancer (SIR 4.57), skin excluding basal and squamous cancer (SIR 1.63), and lung cancer (SIR 1.61). Of hematological malignancies (SIR 4.21), the highest risks were observed for ALL (SIR 10.47), extranodal NHL (SIR 8.46), lymphoma (SIR 6.52), NHL (SIR 6.53), Hodgkin lymphoma (SIR 6.19), nodal NHL (SIR 5.59), AML (SIR 3.77), ANLL (SIR 3.35), myeloid and monocytic leukemia (SIR 3.12), non-lymphocytic leukemia (SIR 2.74), and leukemia (SIR 1.92).

Among patients who developed SM 60-119 months after WM diagnosis, there were elevated risks for solid tumors [mouth cancer (SIR 5.55) and skin cancer (SIR 2.63)], and for hematological malignancies (SIR 5.62), [extranodal NHL (SIR 13.59), NHL (SIR 9.73), lymphoma (SIR 9.32), nodal NHL (SIR 7.76), ANLL (SIR 4.25), AML (SIR 3.38), and non-lymphocytic leukemia (SIR 2.78)]

The greatest risks of development of SM more 120 months after WM diagnosis were observed for male genital organ cancer (SIR 46.42), brain cancer (SIR 6.35), and skin cancer excluding basal and squamous (SIR 2.41). High risks of hematological malignancies (SIR 5.96), were seen for extranodal NHL (SIR 15.50), NHL (SIR 10.34), lymphoma (SIR 9.94), NHL nodal (SIR 7.63), AML (7.50), ANLL (SIR 6.80), myeloid and monocytic leukemia (SIR 5.06), non-lymphocytic leukemia (SIR 4.48), and leukemia (SIR 2.79), (Table 3).

**Table 3.** Standardized incidence ratios of secondary malignancy in Waldenström macroglobulinemia patients, stratified by latency

	Latency			
*Location*	*Total*	*12-59 months*	*60-119 months*	*120+ months*
*All sites*	1.53*	1.46*	1.66*	1.50*
*All sites excluding Non-Melanoma Skin*	1.52*	1.44*	1.65*	1.48*
*All Solid Tumors*	1.12*	*1.12*	1.20*	*0.93*
*Floor of Mouth, and Gum and Other Mouth*	3.28*	*2.72*	5.55*	*0*
*Gum and Other Mouth*	*3.05*	*1.2*	7.16*	*0*
*Digestive System*	0.73*	0.59*	*0.88*	*0.89*
*Colon, Rectum and Anus*	0.65*	0.50*	*0.88*	*0.69*
*Colon and Rectum*	0.65*	0.52*	*0.84*	*0.71*
*Colon excluding Rectum*	*0.75*	0.52*	*1.12*	*0.71*
*Pancreas*	*0.74*	0.27*	*1.18*	*1.33*
*Respiratory System*	1.44*	1.61*	*1.21*	*1.36*
*Lung, Bronchus, Trachea, Mediastinum and Other Resp Org*	1.44*	1.60*	*1.19*	*1.44*
*Lung and Bronchus*	1.44*	1.60*	*1.2*	*1.44*
*Skin excluding Basal and Squamous*	2.10*	1.63*	2.63*	2.41*
*Melanoma of the Skin*	1.88*	*1.3*	2.57*	*2.15*
*Other Non-Epithelial Skin*	4.09*	4.57*	*3.16*	*4.66*
*Breast*	*0.75*	*0.82*	*0.91*	0.17*
*Prostate*	*1.08*	*1.19*	*1.17*	0.32*
*Other Male Genital Organs*	*6.42*	*0*	*0*	46.42*
*Brain and Other Nervous System*	2.63*	*0.85*	3.89*	6.13*
*Brain*	2.73*	*0.88*	4.04*	6.35*
*All Lymphatic and Hematopoietic Diseases*	4.95*	4.21*	5.62*	5.96*
*Lymphoma*	7.98*	6.52*	9.32*	9.94*
*Hodgkin Lymphoma*	*3.29*	6.19*	*0*	*0*
*Hodgkin - Nodal*	*3.41*	6.43*	*0*	*0*
*Non-Hodgkin Lymphoma*	8.19*	6.53*	9.73*	10.34*
*NHL - Nodal*	6.62*	5.59*	7.76*	7.63*
*NHL - Extranodal*	11.30*	8.46*	13.59*	15.50*
*Leukemia*	2.01*	1.92*	*1.81*	2.79*
*Acute Lymphocytic Leukemia*	8.04*	10.47*	*7.86*	*0*
*Non-Lymphocytic Leukemia*	3.02*	2.74*	2.78*	4.48*
*Acute Non-Lymphocytic Leukemia (ANLL)*	4.20*	3.35*	4.25*	6.80*
*Myeloid and Monocytic Leukemia*	3.28*	3.12*	*2.71*	5.06*
*Acute Myeloid Leukemia*	4.22*	3.77*	3.38*	7.50*
*Miscellaneous*	1.82*	1.94*	*1.61*	*1.9*

* represents statistical significance

### Risk of SM stratified by age

An increased risk of SMs was noted for patients aged 50-74 years (SIR 1.75) and over 75 years (SIR 1.3) but not for patients younger than 50. Patients aged 50-74 years had the highest risk of developing nonepithelial skin cancer (SIR 5.57), followed by skin excluding basal and squamous cancer (SIR 3.21), tumors of the brain (SIR 3.32), respiratory cancer (SIR 1.65), and colon and rectum cancer (SIR 0.46). The risk for developing hematological malignancies was also higher (SIR 3.77), extra-nodal NHL (SIR 14.81), NHL (SIR 11.58), lymphoma (SIR 11.08), nodal NHL (SIR 7.42), ANLL (SIR 6.05), AML (SIR 5.94), myeloid and monocytic leukemia (SIR 4.40), non-lymphocytic leukemia (SIR 4.02), and leukemia (SIR 2.28).

Patients aged >75 years had the highest risk of developing SMs of the cervix in females (SIR 8.19), followed by mouth cancer (SIR 4.24), nonepithelial skin cancer (SIR 3.56), and gastrointestinal malignancies (SIR 0.71). The risk for developing hematological malignancies showed SIR 3.77, ALL (SIR 16.64), extra-nodal NHL (SIR 8.91), NHL (SIR 5.82), lymphoma (SIR 5.78), nodal NHL (SIR 4.23), AML (SIR 3.29), myeloid and monocytic leukemia (SIR 2.68), and leukemia (SIR 1.86), (Table 4).

**Table 4.** Standardized incidence ratios of secondary malignancy in Waldenström macroglobulinemia patients, stratified by gender, age, and race.

	*Total*	*Male*	*Female*	*0-49*	*50-74*	*75+*	*White*	*Black*	*Other*
*All sites*	1.53*	1.56*	1.46*	*2.62*	1.74*	1.35*	1.51*	*1.54*	2.32*
*All sites excluding Non-Melanoma Skin*	1.52*	1.55*	1.43*	*2.63*	1.72*	1.34*	1.49*	*1.54*	2.33*
*All Solid Tumors*	1.12*	1.19*	*0.97*	*1.19*	1.23*	*1.03*	1.11*	*0.91*	*1.69*
*Oral Cavity and Pharynx*	*1.66*	*1.52*	*2.19*	*0*	*1.77*	*1.57*	1.75*	*0*	*0*
*Floor of Mouth, and Gum and Other Mouth*	3.28*	*2.78*	*4.31*	*0*	*2.12*	4.24*	3.47*	*0*	*0*
*Gum and Other Mouth*	*3.05*	*1.88*	5.22*	*0*	*1.52*	4.11*	3.23*	*0*	*0*
*Digestive System*	0.73*	0.67*	*0.85*	*0*	*0.76*	0.71*	0.69*	*1.21*	*1.2*
*Colon, Rectum and Anus*	0.65*	*0.66*	*0.64*	*0*	0.46*	*0.78*	0.61*	*1.24*	*1.45*
*Colon and Rectum*	0.65*	*0.64*	*0.67*	*0*	0.48*	*0.77*	0.60*	*1.27*	*1.47*
*Cecum*	*1.47*	*0.91*	*2.28*	*0*	*0*	2.18*	*1.42*	*3.63*	*0*
*Sigmoid Colon*	*1.15*	*0.59*	*0*	*0*	*0.91*	*0.25*	0.15*	*3.87*	*6.26*
*Rectum and Rectosigmoid Junction*	0.37*	*0.39*	*0.32*	*0*	*0.59*	0.18*	*0.4*	*0*	*0*
*Rectum, Rectosigmoid Junction, Anus, Anal Canal and Anorectum*	0.41*	*0.47*	*0.26*	*0*	*0.52*	*0.31*	*0.44*	*0*	*0*
*Respiratory System*	1.44*	1.60*	*1.1*	*8.33*	1.65*	*1.27*	1.43*	*1.66*	*1.56*
*Lung, Bronchus, Trachea, Mediastinum and Other Resp Org*	1.44*	1.62*	*1.09*	*9.61*	1.67*	*1.26*	1.45*	*1.35*	*1.08*
*Lung and Bronchus*	1.44*	1.62*	*1.09*	*9.7*	1.67*	*1.26*	1.46*	*1.35*	*1.09*
*Skin excluding Basal and Squamous*	2.10*	1.96*	2.65*	*0*	3.21*	*1.36*	2.12*	*0*	*0*
*Melanoma of the Skin*	1.88*	1.85*	*1.97*	*0*	3.04*	*1.04*	1.88*	*0*	*0*
*Other Non-Epithelial Skin*	4.09*	2.85*	8.50*	*0*	5.57*	3.56*	4.17*	*0*	*0*
*Cervix Uteri*	*2.96*	*0*	*2.96*	*0*	*0*	8.19*	*3.37*	*0*	*0*
*Urinary Bladder*	*1.08*	*1.14*	*0.69*	*0*	*1.36*	*0.95*	*0.99*	*2.17*	5.24*
*Brain and Other Nervous System*	2.63*	2.56*	*2.79*	*0*	3.19*	*2.15*	2.51*	*0*	*11.45*
*Brain*	2.73*	2.65*	*2.92*	*0*	3.32*	*2.22*	2.60*	*0*	*12.33*
*Endocrine System*	*1.38*	*1.67*	*1.13*	*0*	*2.17*	*0*	*1.48*	*0.16*	*0*
*All Lymphatic and Hematopoietic Diseases*	4.95*	4.66*	5.63*	11.62*	6.72*	3.77*	4.75*	8.43*	8.98*
*Lymphoma*	7.98*	7.44*	9.13*	19.40*	11.08*	5.78*	7.62*	14.32*	16.20*
*Non-Hodgkin Lymphoma*	8.19*	7.63*	9.37*	23.56*	11.58*	5.82*	7.81*	15.36*	16.72*
*NHL - Nodal*	6.62*	6.25*	7.42*	*17.64*	10.03*	4.23*	6.15*	23.83*	12.39*
*NHL - Extranodal*	11.30*	10.40*	13.19*	*35.45*	14.81*	8.91*	11.14*	*0*	23.19*
*Myeloma*	1.93*	*1.76*	*2.33*	*0*	*2.02*	*1.87*	1.96*	*2.22*	*0*
*Leukemia*	2.01*	2.21*	*1.48*	*0*	2.28*	1.86*	1.88*	9.45*	*0*
*Acute Lymphocytic Leukemia*	8.04*	*8.16*	*7.81*	*0*	*0*	16.64*	8.48*	*0*	*0*
*Non-Lymphocytic Leukemia*	3.02*	3.47*	*1.91*	*0*	4.02*	2.52*	2.89*	11.43*	*0*
*Acute Non-Lymphocytic Leukemia (ANLL)*	4.20*	5.33*	*1.44*	*0*	6.05*	3.23*	3.98*	18.21*	*0*
*Myeloid and Monocytic Leukemia*	3.28*	3.71*	*2.19*	*0*	4.40*	2.68*	3.12*	13.21*	*0*
*Acute Myeloid Leukemia*	4.22*	5.29*	*1.61*	*0*	5.94*	3.29*	3.96*	20.12*	*0*
*Mesothelioma*	*2.54*	*2.18*	*5.06*	*0*	*2.19*	*2.69*	*1.96*	*0*	45.97*
*Karposi Sarcoma*	*4.13*	*4.9*	*0*	*0*	*0*	*6.47*	*4.4*	*0*	*0*
*Miscellaneous*	1.82*	1.73*	2.00*	*24.38*	2.72*	*1.39*	1.82*	*1.79*	*1.84*

* represents statistical significance

### Risk of SM stratified by gender

The risk of SM was significantly increased in males (SIR 1.56) and females (SIR 1.46). On subsite evaluation, males had a substantially greater risk of SMs involving nonepithelial skin (SIR 2.85) followed by the brain (SIR 2.65), respiratory cancer (SIR 1.62), skin excluding basal and squamous (SIR 1.96), melanoma of skin (SIR 1.85) and digestive system (SIR 0.67). Their risk for hematological malignancies showed SIR of 4.66 being SIR 7.44 for lymphoma and SIR 2.21 for leukemia. The highest risk was of extra-nodal NHL (SIR 10.40), followed by nodal NHL (SIR 6.35), ANLL (SIR 5.33), AML (SIR 5.29), myeloid and monocytic leukemia (SIR 3.71) and non-lymphocytic leukemia (SIR 3.47). Female patients also showed the highest of SMs involving nonepithelial skin (SIR 8.50), followed by gum and mouth (5.22), skin excluding basal and squamous (SIR 2.56). They had a higher risk for hematological malignancies than males (SIR 5.63). The risk for extra-nodal NHL was (SIR 13.19), NHL (SIR 9.37), lymphoma (SIR 9.13), and NHL Nodal (SIR 7.42). (Table 4)

### Risk of SM stratified by race

The risk of SM was significantly greater in Caucasians (SIR 1.51) and “Other” (American Indian/Alaska Native, Asian/Pacific Islander) (SIR 2.32) but not in African Americans. Among solid tumors, Caucasians presented with the highest incidence of nonepithelial skin cancer (SIR 4.17), followed by mouth cancer (SIR 3.47), brain cancer (SIR 2.60), skin excluding basal and squamous (SIR 2.12), melanoma of the skin (SIR 1.88), respiratory cancer (SIR 1.46), and gastrointestinal cancer (SIR 0.69). Caucasians also had a higher risk of hematological malignancies (SIR 14.750); extranodal NHL (SIR 11.14), ALL (SIR 8.48), NHL (SIR 7.81), lymphoma (SIR 7.62), nodal NHL (SIR 6.15), ANLL (SIR 3.98), AML (SIR 3.96), myeloid and monocytic leukemia (SIR 3.12), non-lymphocytic leukemia (SIR 2.89), myeloma (SIR 1.86), and miscellaneous (SIR 1.82). African Americans were at higher risk of developing hematological malignancies (SIR 8.43): nodal NHL (SIR 23.83), AML (SIR 20.12), ANLL (SIR 18.21), NHL (SIR 15.36), lymphoma (SIR 14.32), myeloid and monocytic leukemia (SIR 13.21), non-lymphocytic leukemia (SIR 11.43), and leukemia (SIR 9.45). Other races were at high risk for developing mesothelioma (SIR 45.97), and urinary bladder malignancies (SIR 5.24), as well as hematological malignancies (SIR 8.98): extranodal NHL (23.19), NHL (SIR 16.72), lymphoma (SIR 16.20), and nodal NHL (SIR 12.39). (Table 4)

## Discussion

WM is an uncommon disease and the precise mechanism of SM development is relatively unclear. Several hypotheses have been explored: genetic predisposition with a family history of WM or plasma cell disorder,^10^ immune dysregulation resulting in heterogeneous histological transformations,^11^ or treatment-targeting related SMs from alkylating agents such as chlorambucil, nucleoside-analogs, and/or proteasome inhibitors.^12,13^ As a result, treating secondary malignancies is especially difficult; prior data have suggested that treated WM patients are at greater risk of secondary cancer development compared to their non-treated counterparts.^14^ This study aimed to analyze the incidence of SM in WM patients, stratifying specific patient characteristics such as age, gender, race, and SM site to identify risk factors.

This is the most recent US population-based analysis assessing the incidence of SM in patients with WM compared to the general population. Our study found that among all 4,112 WM patients who fit the inclusion criteria, 17% (699 patients) were found to have SM. An overall significant increase in risk of SM, secondary primary solid tumor development, and secondary hematological malignancy was seen in patients with WM compared to the general population: SIR 1.53, 1.12, and 4.95, respectively. Although there is a clear association between WM and SM development, only a handful of prior population-based analyses have reported this risk. The findings of this study encourage the implementation of specific screening methods to detect the most common secondary malignancies in WM patients, with respect to site, age, gender, race, and latency. The heightened incidence of skin tumors should prompt regular dermatological screening, including annual skin examination and monthly skin self-examination. Appropriate screening should be pursued for demographic risk factors, specifically in patients aged 50-74 years (SIR 1.75), male (SIR 1.56) and female (SIR 1.46) patients, and Caucasians (SIR 1.51) and American Indian/AK Native, Asian/Pacific Islander (SIR 2.32) patients. Wood *et al.*^15^ have laid out comprehensive screening guidelines for patients with secondary malignancies.

Utilizing data from primary WM Greek patients diagnosed between 1937 and 2007, Philpot *et al.*^16^ noted that 15% of developed a SM, comparable to the 17% of patients reported in our study. Their study noted that the highest SM risk was attributed to acute myeloid leukemia (SIR 5.9) development, followed by myeloma (SIR 4.9), NHL (SIR 4.9), colon cancer (SIR 2.30), and lung cancer (SIR 1.7). This contrasted with our study, where the highest risk of SM was seen in extra-nodal NHL (SIR 11.3), NHL (SIR 8.29), lymphoma (SIR 7.98), and nodal HL (SIR 6.62). Of note, our study also supported the data reported by Philpot *et al*., in which the lowest SM risk was in gastrointestinal cancers (SIR 0.73) and respiratory cancers (SIR 1.44).

Although Varettoni et al.’s study^14^ did not stratify the risk of SM development by age, gender, or race, it explored these characteristics through an age and gender-matched control population in Italy. Fourteen percent of patients developed SM, with an overall 1.69 times significantly higher risk than in the general population. Of note, no significant difference in SM was found between males and females (p=0.67), which conflicted with the findings of our study, although no difference in the type of SM was detected in each. In addition, no significant difference was noted due to age (p=0.91), which was similar to our reported findings. Varettoni *et al.* also reported a significantly higher risk of SM for diffuse large B-cell lymphoma (SIR 9.24), supported by our study, followed by MDS/AML (SIR 9.24), and brain cancer (SIR 8.05).^16^

Giri *et al.*^17^ published the first US population-based study in 2015, including patients diagnosed with WM between 1992 and 2010. They reported that 15.3% of patient, at a median age of 74 years, developed SM with a significant SIR of 1.49, and AER of 97.01 per 10,000 population. Our study included patients between 2000 and 2018, reporting higher values: 17% of patients developing SM, with a mean age of 74, a significant SIR of 1.53, and AER of 102.69 per 10,000 population. Giri *et al.* reported the highest risk of SM involving NHL (SIR 4.96), and the lowest of primary lung tumors (SIR 1.6), similar to our findings. However, we found gastrointestinal cancers to exhibit the lowest risk (SIR 0.73). The SIR was identical when stratified according to gender, and although a higher risk was noted in the >60 age group, it was comparable when stratified according to age.^17^

Although their results were similar to those of Varettoni *et al.*,^14^ McMaster *et al.*^18^ reported a higher risk of secondary cancer in younger patients (<65) when analyzing the WM SEER registry data between 1992 and 2011. Castillo *et al.*^19^ analyzed data from the same 20-year period and supported a higher risk of SM development (both solid and hematological tumors) in patients younger than 65 years of age (SIR 2.24). This difference was particularly significant for solid tumors (SIR 1.63 versus SIR 1.13) and hematological malignancies (SIR 9.04 versus SIR 3.62) in younger than in older patients. Compared to all these studies, our analysis demonstrated an increased risk of SM for patients aged 50-75 (SIR 1.75) compared to patients younger than 50.

Castillo *et al.* also noted a significantly higher risk of secondary hematological malignancies in females than males (SIR 5.82 versus 3.43), similar risk of solid tumors in both genders, and comparable risks when stratified according to race.^19^ Compared to the studies mentioned above, our analysis demonstrated a significant higher risk of SM in Caucasians (SIR 1.51) and American Indian/AK Native, Asian/Pacific Islander (SIR 2.32) patients, but not in African Americans. Further, in our study, Caucasian patients had a higher risk of SM involving hematological malignancies (SIR 14.75) than African Americans (SIR 8.43). Castillo *et al.*^19^ presented the last US population-based study published in 2015, concluding that WM patients had a 49% higher risk of SM than the general population, with updated results from an earlier study reporting a higher value of 53%.^20^

The last reported population-based study in 2018 by Gavriatopoulou *et al.*^21^ analyzed all symptomatic WM patients registered within the Greek Myeloma Study Group and found that among 598 patients, 7.7%, developed a SM, with an incidence rate of 1 per 100 patients per year and a median age of 69 years. This study found a lower risk of SM development compared to the 15% in the previous study involving Greek patients reported in 2011.^16^ Unlike Philpot *et al.*,^16^ Gavriatopoulou *et al*. reported risk-stratified results according to gender, with a significantly higher incidence ratio in males (0.5), and no significant difference noted between younger and older patients >75.^20^ The aforementioned data provide context to the historical trends of SM in WM and underscores the need for aggressive screening and treatment.

A major limitation of our study was the lack of data on variables such as genetic predisposition and cancer risk factors, such as smoking status and obesity, which may influence the study results. The lack of detailed treatment data was another limitation of our study, preventing us from analyzing the effect of treatment modalities which may alter the data regarding SM incidence and outcome. Another limitation was a possible overestimation of SM cases due to the misclassification of metastasis of primary tumors as SM. Despite this, we aimed to reduce the bias arising from the mentioned limitation by excluding patients diagnosed with an SM appearing at least a year after WM diagnosis.

### Author contributions

Conceptualization and analysis design: MEUR, MSF, FA; Acquisition and interpretation of data: MEUR, MH, ZS, OSA, RA, FF; drafting the paper and critical revision: MEUR, MH, ZS, OSA, RA, FF, JB, AI; approval of the submitted and final versions: all authors.
